# Chronic Invasive Fungal Rhinosinusitis in Immunocompetent Patients: A Retrospective Chart Review

**DOI:** 10.3389/fsurg.2020.608342

**Published:** 2020-12-16

**Authors:** Naif H. Alotaibi, Omar Abu Omar, Mays Altahan, Haifa Alsheikh, Fawziah Al Mana, Zeyad Mahasin, Eyas Othman

**Affiliations:** ^1^Department of Otolaryngology, Head and Neck Surgery and Communication Sciences, King Faisal Specialist Hospital & Research Center, Riyadh, Saudi Arabia; ^2^College of Medicine, Alfaisal University, Riyadh, Saudi Arabia

**Keywords:** chronic invasive fungal rhinosinusitis, immunocompetent patients, chronic rhino sinusitis, functional endoscopic sinonasal surgery (FESS), anti-fungal

## Abstract

**Objective:** We report cases of Chronic Invasive Fungal Sinusitis (CIFS) in patients considered as immunocompetent at tertiary care center (King Faisal Specialist Hospital), to analyze their clinical, biological, radiological features, and management.

**Material and methods:** A retrospective chart review of CIFS in immunocompetent patients. The inclusion criteria as the following: immunocompetent patients of any age with histopathological findings of CIFS. Immunocompromised patients, acute Invasive Fungal Sinusitis (IFS), non-invasive fungal rhinosinusitis, and no positive histological findings were excluded.

**Results:** Seventeen (17) patients were included. The species isolated included: Aspergillus (most frequent) & Mucor. Surgical treatment approaches were described. Complications reported include CSF leak, blindness, recurrence, and death.

**Conclusions:** Early diagnosis and management of CIFS improve clinical outcomes.

## Introduction

Chronic Invasive Fungal Sinusitis (CIFS) is a sub-entity of fungal rhinosinusitis, with histopathological evidence of fungal invasion to the soft tissue of nasal and paranasal sinuses. Invasive fungal sinusitis (IFS) mostly affects immunocompromised patients, including patients with diabetes or solid organ transplant recipients, which has a rapid, aggressive course. CIFS classically presents in known healthy individuals, unlike Acute Invasive Fungal Sinusitis (AIFS), which usually arises in immunocompromised hosts. The disease, CIFS, often progresses gradually over weeks or months. The absence of apparent predisposing factors can lead to a diagnostic dilemma and delay in surgical or medical interventions. Nasal obstruction, facial pain, rhinorrhea, headaches, and epistaxis are non-specific symptoms that are repeatedly seen in patients with CIFS. If neglected and kept untreated, CIFS can lead to invasion to neighboring structures and possible medical manifestations, including proptosis, altered mental status, seizures, and intracranial complications (1, 2).

In CIFS, chronic symptoms and signs in immunocompetent patients might mimic other types of chronic sinusitis resulting in ideal treatment delay or misdiagnosis. The CIFS in immunocompetent patients require a better understanding of better treatment. Limited studies have been conducted concerning CIFS in immunocompetent hosts, and a small number of cases have been reported in the literature.

CIFS can be categorized into granulomatous and non-granulomatous forms, based mainly on the presence of submucosal granulomatous inflammation, with fungal hyphae and extensive fibrosis (3–5).

## Methods

At King Faisal Specialist Hospital (KFSH), Otorhinolaryngology department, medical records were abstracted for patients with a diagnosis of Chronic Invasive Fungal Rhinosinusitis (CIFS) (a retrospective chart review of CIFS) with the following inclusion: to be immunocompetent and had no prior history of immunosuppressive therapy or comorbidities (by reviewing medical records of each included patient). Data regarding demographics, ENT/systemic medical histories, clinical variables, therapy initiations, and reasons for changes, adverse events, and resource utilization were recorded from chosen eligible charts. Those patients were selected as they met the inclusion criteria that were set. Immunocompetent patients of any age group with histopathological findings of CIFS were included. Immunocompromised patients with acute, non-invasive fungal rhinosinusitis, and no positive histological findings were excluded. This chart review is an update of previously published data by Al Rajhi et al. (6).

Data were analyzed by using “SPSS” (IBM Corp. Released 2013. IBM SPSS Statistics for Windows, Version 21.0. Armonk, NY: IBM Corp.)

The study was approved by the office of research affairs at King Faisal Specialized Hospital & Research Center (KFSH&RC) (ORA#2181 164).

## Results

Seventeen (17) patients met the inclusion criteria. The patients had a mean age of 20 years (range 4–33). 58.8% were female (10 of 17), and 41.2% were men (seven of 17). The main presenting symptoms at our tertiary center are listed in Table 1.

**Table 1:** The presenting symptoms of patients at the tertiary care center in a descending order.

**Symptoms**	**Number of patients (*n*)**	**Percentage (%)**
Nasal congestion	11	64.7
Proptosis	11	64.7
Vision loss	7	41.2
Rhinorrhea	6	35.3
Facial swelling	3	17.6
Epistaxis	3	17.6
Subjective anosmia	2	11.8
Fever	2	11.8
Subjective hyposmia	2	11.8
Blurred vision	1	5.9

29.4% of the patients referred for care were diagnosed with Asthma (five patients out of 17). On examination, 52.9% (9) patients had nasal polyps, 5.9% (1) patient had unilateral orbital abscess that needed surgical drainage, and 23.5% (4) patients presented with palpable cervical lymph nodes.

Seven (7) patients had pansinusitis, including two (2) with skull base involvement, one (1) isolated frontal, and one (1) isolated sphenoidal Chronic Invasive Fungal Sinusitis (CIFS). The rest were from multiple sinuses. One (1) patient who had skull base extension required craniotomy after Functional Endoscopic Sinus Surgery (FESS). Both patients with skull base extension presented with vision loss. After surgical and medical management (both had three (3) surgeries), one (1) of the two (2) patients had irreversible blindness after four (4) years of follow up.

All patients underwent Computed Tomography (CT) scan of paranasal sinuses as part of their medical investigation plan (100%; 17). The majority of included patients (82.4%; 14) underwent Magnetic Resonance Imaging (MRI) to assess pathology characteristics, involved sinuses, and disease extension.

The majority of patients had surgical treatment (94.1%; 16), of which 23.5 % (4) patients had both open and endoscopic approaches, 11.8% (2) patients had only open approach to debride and clear the paranasal sinuses from fungal infections. Functional Endoscopic Sinus Surgery (FESS) as an only surgical approach was performed in 58.8% (10) patients. 82.4% (14) patients had combined medical and surgical treatment; the medical treatment was oral and/or intravenous anti-fungal therapy. One (1) patient had only medical therapy because his condition was advanced and was unfit for a surgical procedure.

The mean follow-up period was 46.6 months (std 68.5), for which most patients recovered from the disease (94.1%; 16), and only one (1) mortality caused by the advanced stage of the disease with intracerebral extension at presentation to our institution.

The histopathology assessment demonstrated 35.3% (6) patients with granulomatous type, 64.7% (11) patients diagnosed as Chronic Invasive Fungal Sinusitis. Fungal cultures isolated were as the following: Aspergillus Falvus 58.8% (10), Mucor 11.8% (2) (1 patient had both aspergillus flavus and Mucor), Aspergillus Fumigatus: 5.9% (1), and four (4) were untraceable.

## Discussion

Fungi are widespread airborne eukaryotic organisms that, in specific settings, can inflict morbidity in the upper airway tract, one of which is fungal rhinosinusitis (7). Fungal rhinosinusitis occurs when the fungus invades the nasal and sinus cavities due to favorable situations, including the index of pathogenicity of fungal species, the immunological status of the host, and overall complex cellular and humoral responses (7). Fungal sinusitis severity ranges from non-invasive (fungal ball, Allergic fungal sinusitis) to invasive acute or chronic fungal infections. Chronic fungal infections are prevalent among both immunosuppressed and immunocompetent hosts, unlike Chronic Invasive Fungal Sinusitis (CIFS) which typically presents in immunocompetent patients (4).

Researchers consider invasive fungal sinusitis to be a progressively prevailing disease, yet still a rare infectious disease preceded by progressive tissue destruction, angioinvasion, and mostly, unfortunate outcomes. CIFS classically presents in known healthy individuals, unlike Acute Invasive Fungal Sinusitis (AIFS), which usually arises in immunocompromised hosts. CIFS often progresses gradually over weeks or months. Absence of apparent predisposing factors or lack of accurate recognition can lead to a diagnostic challenge and delay in any surgical or medical interventions (8–10). It is believed and also highlighted in our study results, that findings including nasal obstruction, facial pain, rhinorrhea, headaches, and epistaxis are non-specific symptoms that are repeatedly seen in patients with CIFS (10). If neglected and kept untreated, CIFS can lead to invasion of neighboring structures and possible medical manifestations, including proptosis, altered mental status, seizures, and intracranial complications (1, 2).

CIFS is rare, unlike in Africa, Sudan, and India, where it is believed to be more prevalent (4, 11–14). As previously mentioned, CIFS can be categorized into granulomatous and non-granulomatous forms. *Aspergillus flavus is the most common causative organism in most cases of granulomatous and non-granulomatous CIFS, which also matches the findings of this paper's results* (11, 15). Although *A. flavus* were the most frequent causative species in this study, we found in this study two patients with Mucor species, one of them was identified in addition to *A. Flavus* (young female); our findings support what already exists in the literature; the most common isolated fungi were from Aspergillus species. Our findings also supported what was published by local colleagues Kameswaran et al. (16) and by primary study from the same center (6), it is important to highlight that this study included all causative fungal species, the previous study from our center included only patients with chronic invasive aspergillosis of the paranasal sinuses in immunocompetent hosts. The second frequent causative fungi isolated in this series was Mucor. Aspergillus and Mucor fungal are believed to be the common organisms causing CIFS (17).

When compared to other forms of chronic rhinosinusitis, a limited number of studies have been conducted concerning CIFS in immunocompetent hosts, and a small number of cases have been reported in the literature. In this retrospective chart review, we report seventeen (17) new cases of CIFS in patients considered as immunocompetent at King Faisal Specialist Hospital & Research Center (KFSH&RC), to analyze their clinical, biological, radiological features and management.

Proptosis and nasal obstruction were the most frequent presenting symptoms at the presentation to our tertiary center; it similar to previous findings by primary study of the same center (6) and by Mehta et al. (18). We believe that this serious disease stage is related to the ambiguity of CIFS in immunocompetent patients at its early stage, which might be treated as chronic sinusitis before the final diagnosis is reached.

Although most patients underwent surgery as part of the treatment plan (94.1%; 16), the surgical approach was variable between open, endoscopic, and combined. It is essential to highlight that KFSH&RC is considered amongst the main referral centers in the Kingdom of Saudi Arabia (KSA), receiving a nationwide referral within KSA. Not limited to this reason, patients might present with an advanced stage of CIFS; this might be explained as well for the highest sinus involved in this series, frontal sinus (counting isolated or involved in pansinusitis), rather than other sinuses repeatedly reported to the commonest affected sinuses: maxillary and ethmoid (17–19).

With a relatively long mean follow up period of 46 months, one (1) patient passed away because of the advanced stage of the disease. Generally, this study results that combined surgical and medical anti-fungal therapy, and repeated surgery for debridement -if necessary- plays a significant role in better outcomes.

## Conclusions

CIFS often progresses gradually over weeks or months. Absence of apparent predisposing factors or lack of early accurate recognition can lead to a diagnostic challenge and delay in any surgical or medical interventions. Early diagnosis and management of CIFS improve clinical outcomes. Complete excision of infected paranasal tissues if possible is essential to control the disease, the treatment plan is might differ and tailored according to disease severity, extension and patient's medical condition.

## Data Availability Statement

The raw data supporting the conclusions of this article will be made available by the authors, without undue reservation.

## Ethics Statement

The studies involving human participants were reviewed and approved by the office of research affairs at King Faisal Specialized Hospital & Research Center (KFSH&RC) (ORA#2181 164). The patients/participants provided their written informed consent to participate in this study.

## Author Contributions

NA: conceived of the presented idea, contributed to the design, implementation of the research, to the analysis of the results, and to the writing of the manuscript. OO: contributed to the design, implementation of the research, to the analysis of the results, and to the writing of the manuscript. MA: acquisition of data. HA, FA, and EO: contributed to the analysis of the results and to the writing of the manuscript. ZM: contributed to the design and implementation of the research. All authors contributed to the article and approved the submitted version.

## Conflict of Interest

The authors declare that the research was conducted in the absence of any commercial or financial relationships that could be construed as a potential conflict of interest.

## References

[1] Thurtell M Chiu A Goold L Akdal G Crompton J Ahmed R. Neuro-ophthalmology of invasive fungal sinusitis: 14 consecutive patients and a review of the literature. Clin Exp Ophthalmol. (2013) 41:567–76. 10.1111/ceo.12055 23279383

[2] Vakharia K Durand M Hamilos D Geyer J Holbrook E. An atypical case of chronic invasive fungal sinusitis and its management. Otolaryngology. (2010) 142:150–1. 10.1016/j.otohns.2009.06.743 20096246

[3] Challa S Uppin S Hanumanthu S Panigrahi M Purohit A Sattaluri S. Fungal rhinosinusitis: a clinicopathological study from South India. Eur Archiv Oto-Rhino-Laryngol.(2010) 267:1239–45. 10.1007/s00405-010-1202-6 20107998

[4] Montone K. Pathology of fungal rhinosinusitis: a review. Head Neck Pathol. (2016) 10:40–6. 10.1007/s12105-016-0690-0 26830404 PMC4746136

[5] Michael J Ashbee R Mathews M Michael R. Mycological profile of fungal sinusitis: an audit of specimens over a 7-years period in a tertiary care hospital in Tamil Nadu. Ind J Pathol Microbiol. (2008) 51:493. 10.4103/0377-4929.43738 19008573

[6] Alrajhi AA Enani M Mahasin Z Al-Omran K. Chronic invasive aspergillosis of the paranasal sinuses in immunocompetent hosts from Saudi Arabia. Am J Trop Med Hygiene. (2001) 65:83–6. 10.4269/ajtmh.2001.65.83 11504413

[7] Soler Z Schlosser R. The role of fungi in diseases of the nose and sinuses. Am J Rhinol Allergy. (2012) 26:351–8. 10.2500/ajra.2012.26.3807 23168148 PMC3904040

[8] Peral-Cagigal B Redondo-Gonzalez L Verrier-Hernandez A Invasive maxillary sinus aspergillosis: a case report successfully treated with voriconazole and surgical debridement. J Clin Exp Dentistry. (2014) 571:e448–51. 10.4317/jced.51571 PMC4282918 25593673

[9] Clancy C Nguyen M. Invasive sinus aspergillosis in apparently immunocompetent hosts. J Infection. (1998) 37:229–40. 10.1016/S0163-4453(98)91921-1 9892526

[10] Pekala K Clavenna M Shockley R Weiss V Turner J. Chronic invasive fungal sinusitis associated with intranasal drug use. Laryngoscope. (2015) 125:2656–9. 10.1002/lary.25429 26153255 PMC4725714

[11] Stringer S Ryan M. Chronic invasive fungal rhinosinusitis. Otolaryngol Clin North America. (2000) 33:375–87. 10.1016/S0030-6665(00)80012-2 10736411

[12] Thompson G Patterson T. Fungal disease of the nose and paranasal sinuses. J Allergy Clin Immunol. (2012) 129:321–6. 10.1016/j.jaci.2011.11.039 22206776

[13] Chakrabarti A Denning D Ferguson B Ponikau J Buzina W Kita H Fungal rhinosinusitis. Laryngoscope. (2009) 119:1809–18. 10.1002/lary.20520 19544383 PMC2741302

[14] Chakrabarti A Das A Panda N. Controversies surrounding the categorization of fungal sinusitis. Med Mycol. (2009) 47:S299–308. 10.1080/13693780802213357 18663658

[15] Deshazo R. Syndromes of invasive fungal sinusitis. Med Mycol. (2009) 47:S309–14. 10.1080/13693780802213399 18654920

[16] Kameswaran M Al-Wadei A Khurana P Okafor B. Rhinocerebral aspergillosis. J Laryngol Otol. (1992) 106:981–5. 10.1017/S0022215100121528 1479276

[17] Deutsch PG Whittaker J Prasad S. Invasive and non-invasive fungal rhinosinusitis—a review and update of the evidence. Medicina. (2019) 55:319. 10.3390/medicina55070319 31261788 PMC6681352

[18] Mehta R Panda N Mohindra S Chakrabarti A Singh P. Comparison of efficacy of amphotericin B and itraconazole in chronic invasive fungal sinusitis. Ind J Otolaryngol Head Neck Surg. (2012) 65:288–94. 10.1007/s12070-011-0444-y 24427663 PMC3738786

[19] Chopra H Dua K Malhotra V Gupta RP Puri H. Invasive fungal sinusitis of isolated sphenoid sinus in immunocompetent subjects. Mycoses. (2006) 49:30–6. 10.1111/j.1439-0507.2005.01170.x 16367816

